# Genetic Origins of Birth Defects Revealed by New Animal Model

**DOI:** 10.1371/journal.pbio.1001180

**Published:** 2011-10-25

**Authors:** Janelle Weaver

**Affiliations:** Freelance Science Writer, Glenwood Springs, Colorado, United States of America

**Figure pbio-1001180-g001:**
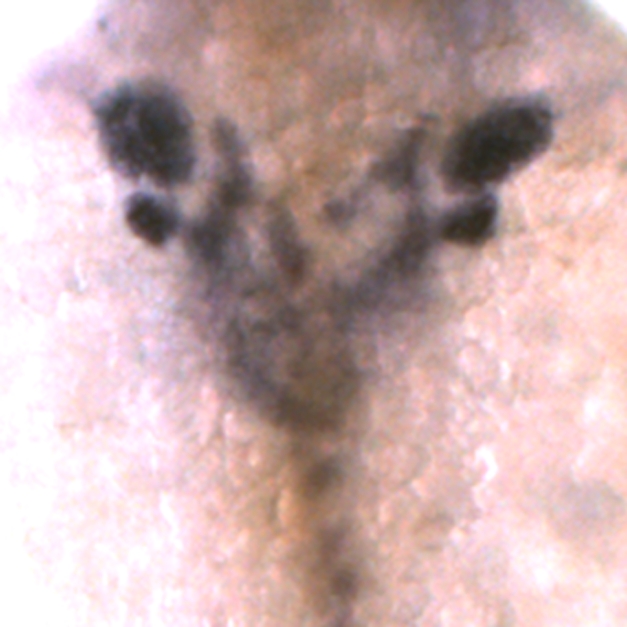
Gut bifurcation, with mirror-image duplication of liver and pancreas, are among the phenotypes of *nipbl*-deficient fish embryos reproduced by combined knockdown of two Nipbl-downstream genes, *sox17* and *foxa2*.

[Fig pbio-1001180-g001]Cornelia de Lange syndrome is a rare congenital disorder marked by malformations of many different body parts, including the face, head, heart, and intestines. Most cases are caused by mutations that reduce levels of the Nipped-B-like (NIPBL) protein, whose best known role is to help ensure that chromosomes separate properly during cell division through its interactions with a protein complex called cohesin. Even a slight decrease in the amount of NIPBL can cause deformities in humans, and an ongoing puzzle in this and other diseases is how such a subtle change in the levels of one protein can give rise to devastating effects.

Given that Nipbl regulates the expression of hundreds of genes, a minor deficiency in this protein could result in many small changes that interact to produce severe and widespread defects. Zebrafish (*Danio rerio*) is a convenient model organism for testing this possibility because its transparent body during early development allows scientists to examine its internal anatomy.

As reported in this issue of *PLoS Biology*, a team led by Arthur Lander and Thomas Schilling, developmental biologists at the University of California at Irvine, has established zebrafish as an animal model of Cornelia de Lange syndrome. The researchers found that a shortage of Nipbl in zebrafish embryos leads to modest declines in the expression of genes involved in organ development, resulting in malformations that characterize the disorder. This finding provides an alternative to single-gene models commonly used to explain a variety of birth defects.

The researchers first searched the zebrafish genome database and identified two genes that code for proteins similar to human NIPBL. They then blocked the production of these proteins, Nipbla and Nipblb, in zebrafish embryos shortly after fertilization.

The loss of both proteins led to various heart and gut abnormalities about one to two days later. The heart and the gut did not form normal loops in most embryos, and the co-occurrence of these defects suggests that they originated from shared deficiencies in the arrangement of organ parts. In support of this idea, several genes involved in creating asymmetric patterns in organs were found to be expressed at low levels and on the wrong side of the body. In addition, many other embryos lacked both an intact heart tube and a complete gut tube.

Before these anomalies appeared, Nipbl depletion reduced the expression of genes required for the formation of the endoderm, a cell layer that gives rise to the gastrointestinal tract. These genes included *sox17*, *foxa2*, *sox32*, and *gata5*. When the researchers partially restored the expression of either *gata5* or *sox32*, the heart tube fused correctly and the entire gut tube formed, indicating that these abnormalities arose from impairments in endoderm development.

Some cases of Cornelia de Lange syndrome are caused by mutations in genes that encode cohesin subunits. Because a loss of Nipbl could also interfere with cohesin activity, the researchers tested whether impaired cohesin function could account for the anomalies seen in Nipbl-depleted embryos. When they lowered the expression of cohesin subunit genes in a new batch of embryos, the expression of *sox32*, *sox17*, and *foxa2* did not change. This result suggests that Nipbl and cohesin regulate distinct sets of genes, and that the disorder is not caused simply by a decrease in cohesin activity.

Taken together, the findings reveal that Nipbl controls a network of genes that contribute to endoderm development and organ asymmetry, and to deformities resembling those of Cornelia de Lange syndrome. Because the gene interactions seen in this condition share features with those presumed to underlie many different genetic disorders, this research may provide new insights into a range of human birth defects.

**Muto A, Calof AL, Lander AD, Schilling TF (2011) Multifactorial Origins of Heart and Gut Defects in** ***nipbl*** **-Deficient Zebrafish, a Model of Cornelia de Lange Syndrome. doi:10.1371/journal.pbio.1001181**

